# Effects of Vitamin D on Epigenetics, Seasonality, and Management of Rheumatoid Arthritis

**DOI:** 10.3390/nu18142359

**Published:** 2026-07-18

**Authors:** Orlando Izzo, Emanuele Gotelli, Elvis Hysa, Rosanna Campitiello, Sabrina Paolino, Carmen Pizzorni, Stefano Soldano, Alberto Sulli, Vanessa Smith, Maurizio Cutolo

**Affiliations:** 1 Laboratory of Experimental Rheumatology, Division of Rheumatology, Department of Internal Medicine and Medical Specialties (Di.M.I.), University of Genova, 16132 Genova, Italy; orlando.izzo@edu.unige.it (O.I.); emanuele.gotelli@unige.it (E.G.); elvis.hysa@edu.unige.it (E.H.); campitiellorosanna@gmail.com (R.C.); sabrina.paolino@unige.it (S.P.); carmen.pizzorni@unige.it (C.P.); stefano.soldano@unige.it (S.S.); albertosulli@unige.it (A.S.); 2 Department of Experimental Medicine (DIMES), University of Genova, 16132 Genova, Italy; 3 AOM—IRCCS Ospedale Policlinico San Martino, 16132 Genova, Italy; 4 Department of Rheumatology, Ghent University Hospital, University of Ghent, 9000 Ghent, Belgium; vanessa.smith@ugent.be; 5 Department of Internal Medicine, Ghent University Hospital, University of Ghent, 9000 Ghent, Belgium; 6 Unit for Molecular Immunology and Inflammation, Flemish Institute for Biotechnology, Inflammation Research Center, 9052 Ghent, Belgium

**Keywords:** vitamin D, rheumatoid arthritis, epigenetics, circannual rhythms, therapy

## Abstract

**Background and Objectives:** Rheumatoid arthritis (RA) is a chronic systemic autoimmune disease in which persistent synovial inflammation and joint damage are influenced not only by immune dysregulation but also by environmental, genetic, and epigenetic factors. Vitamin D is a secosteroid hormone and has emerged as a key immunomodulatory hormone, with reported effects on innate and adaptive immune responses, with potential relevance to RA clinical activity and treatment. This narrative review synthesizes mechanistic and clinical evidence enlightening vitamin D’s immunomodulatory role in RA pathogenesis and management. **Methods:** A comprehensive literature search was carried out on PubMed and MEDLINE databases using Medical Subject Headings (MeSH) terms: “Vitamin D”, “Cholecalciferol”, “Arthritis, Rheumatoid”, “Seasons”, “Epigenomics”, “DNA Methylation”, and “Therapy”. The narrative review highlights evidence published mainly in the last 5 years on the link between vitamin D and RA, focusing on epigenetic interactions, circannual rhythms, and therapeutic implications. **Results:** Emerging data suggest that vitamin D-related epigenetic mechanisms (e.g., DNA methylation, histone acetylation, and microRNA regulation) and genetic polymorphisms have been associated with disease susceptibility and treatment outcomes. Latitude and seasonal fluctuations in serum 25-hydroxyvitamin D levels correlate with variations in RA disease activity, although results remain heterogeneous across studies. Overall, the available evidence supports an association between vitamin D deficiency and greater RA disease activity, while its adequate supplementation has been associated with improvements in inflammatory markers and selected clinical outcomes, especially when tailored to baseline status and individual risk factors, such as limited dietary intake and sunlight exposure. **Conclusions:** Current evidence emphasizes the need for further studies using standardized methods and larger, geographically diverse cohorts to define how best to leverage seasonal vitamin D variations in RA management, considering also the range of concomitant epigenetic modifiers that may influence the effects of vitamin D on the management of RA patients.

## 1. Introduction

Rheumatoid arthritis (RA) is a chronic and systemic autoimmune disease characterized by persistent synovial inflammation, progressive joint destruction, and significant extra-articular manifestations [1]. Despite major advances in the development of targeted synthetic and biologic disease-modifying antirheumatic drugs (DMARDs), a substantial proportion of patients fail to achieve sustained remission or experience adverse effects from long-term immunosuppression [2]. Consequently, there is a growing interest in identifying modifiable environmental (i.e., epigenetic) and hormonal factors associated with disease pathogenesis that could serve as adjunctive therapeutic targets. Among these, vitamin D has been increasingly investigated as a pivotal linker bridging environmental exposure, genetic susceptibility, and immune regulation [3,4].

Recent evidence points to a complex interplay involving epigenetic reprogramming, genetic polymorphisms in the vitamin D pathway, and interactions with the gut microbiota [5,6,7]. Epigenetics encompasses heritable changes in gene expression that do not involve alterations in the underlying DNA sequence, including DNA methylation, histone modifications, and non-coding RNA regulation [8]. DNA methylation, through the addition of methyl groups to cytosine residues in CpG dinucleotides, represents a critical epigenetic mechanism regulating gene expression in RA [9,10].

Epidemiological observations have long noted a higher prevalence of vitamin D deficiency in RA patients compared to the general population, with low serum 25-hydroxyvitamin D [25(OH)D] levels correlating with increased disease activity, higher inflammatory markers, and greater functional disability [1,11].

Moreover, in temperate and higher latitudes, ultraviolet B (UVB) radiation is dramatically reduced during winter months, with complete absence of sufficient UVB for vitamin D synthesis in some northern regions during winter. Accordingly, serum 25(OH)D concentrations exhibit pronounced circannual rhythms, typically peaking in late summer or early fall (August–September) following maximal cumulative sun exposure, and reaching nadir levels during late winter or early spring (February–March) [12,13]. Concomitantly, seasonal changes in RA symptoms have been observed, with spring (after winter) associated with worsening inflammatory symptoms, whilst autumn (after summer) with symptom improvement [13,14].

While randomized controlled trials on vitamin D supplementation in RA have yielded heterogeneous results, emerging meta-analyses suggest that adequate repletion, particularly with personalized dosing strategies, is linked to a reduction in disease activity scores and inflammatory markers [15,16]. This narrative review aims to synthesize current evidence regarding vitamin D’s multifaceted role in RA pathogenesis and management, with particular emphasis on the new findings on epigenetic mechanisms and seasonal variations in vitamin D levels and RA disease activity.

## 2. Methods

A narrative literature search was conducted on PubMed and MEDLINE databases using Medical Subject Heading (MeSH) terms and free-text keywords, including “Vitamin D”, “Cholecalciferol”, “Arthritis, Rheumatoid”, “Seasons”, “Epigenomics”, “DNA Methylation”, and “Therapy”, combined using Boolean operators (AND/OR).

Articles were selected based on their relevance to the topic, including original research articles, systematic reviews, and meta-analyses. All identified publications were independently assessed by the authors through a qualitative appraisal based on their methodological quality, relevance to the objectives of the review, and the originality and scientific significance of their findings.

No restrictions on publication date were applied during the literature search to ensure comprehensive coverage of the available evidence. However, greater emphasis was given to studies published within the last five years (until December 2025) to capture the most recent developments in the field, while earlier landmark studies were included in the narrative review whenever considered essential for contextualizing the evidence.

The reference lists of all eligible articles were also manually screened to identify additional relevant studies that had not been retrieved through the electronic database search.

No language restrictions were initially applied, although the final synthesis focused on English-language publications. Conference abstracts, editorials, and case reports were excluded from the review.

## 3. Vitamin D as Immunomodulatory Hormone

**Figure 1:**
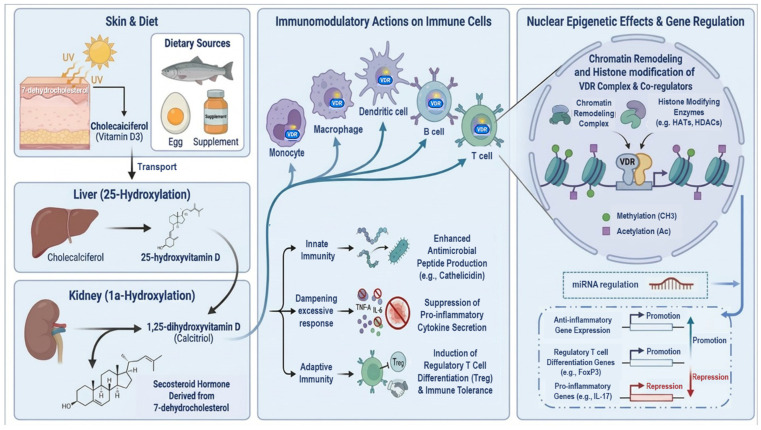
Immunomodulatory and endocrine role of vitamin D in rheumatoid arthritis: genomic and epigenetic effects. Abbreviations: VDR: vitamin D receptor; TNF-α: tumor necrosis factor-alpha; IL-6: interleukin 6; HATs: histone acetyltransferases; HDAC: histone deacetylases; CH_3_: methylation; Ac: acetylation; miRNA: microRNA; FoxP3: forkhead box P3; IL-17: interleukin 17. *Realized with Microsoft^®^ PowerPoint^®^ for Microsoft 365 MSO (Version 2604 Build 16.0.19929.20172).*

Vitamin D belongs to the family of secosteroids, derived from cholesterol (7-dehydrocholesterol) and characterized by a broken B-ring structure [17] (Figure 1). The two main biologically relevant forms are vitamin D_3_ (cholecalciferol), synthesized in the skin through UVB radiation and present in animal-derived foods, and vitamin D_2_ (ergocalciferol), obtained mainly from vegetables and fungi [18,19,20].

After synthesis or ingestion, vitamin D undergoes its first hydroxylation in the liver, catalyzed by 25-hydroxylase (CYP2R1), a cytochrome P450 superfamily member, producing 25-hydroxyvitamin D (25(OH)D), called calcidiol. This metabolite is the predominant circulating form and is commonly used as a biomarker of vitamin D status [17,21]. A second hydroxylation occurs mainly in the kidney by 25(OH)D-1α-hydroxylase (CYP27B1), generating 1,25(OH)_2_D, also named calcitriol, which is the biologically active form with high affinity for the vitamin D receptor (VDR) [22,23].

The chemical structure of 1,25(OH)_2_D allows it to enter cell and nuclear membranes and interact with VDR, which is ubiquitously expressed in immune cells, including monocytes, macrophages, dendritic cells, and B and T lymphocytes [18,19]. Through this receptor, vitamin D can modulate both innate and adaptive immunity, promoting antimicrobial defense while simultaneously dampening excessive pro-inflammatory responses and fostering immune tolerance [24,25].

## 4. The Interaction of Vitamin D with the Immune Cells in RA

Vitamin D is regarded as a hormone associated with the modulation of both innate and adaptive immune responses in autoimmune diseases, including RA [24]. In innate immunity, vitamin D is involved in antimicrobial activity by promoting the production in vitro of microbicidal peptides, including cathelicidin and defensins, while fostering macrophage polarization to an anti-inflammatory (M2) phenotype and modulating the STAT1/TREM-1 pathway and dendritic cell maturation toward a more tolerogenic fate [20,26,27,28]. In adaptive immunity, vitamin D has been shown to suppress excessive pro-inflammatory Th1 and Th17 responses and to promote the expansion of regulatory B and T cells [29].

Specifically, vitamin D has been reported to influence gene expression of pro-inflammatory molecules like interleukin (IL)-1, IL-8, IL-12, tumor necrosis factor alpha (TNF-α), interferon-γ, and toll-like receptors (TLR)-2 and 4 to anti-inflammatory cytokines such as IL-4, IL-5, and IL-10 [25,30].

Through these combined actions, vitamin D may be involved in maintaining immune homeostasis, reducing chronic inflammation, and preventing dysregulated immune responses. Experimental and observational studies suggest that inadequate vitamin D signaling may correlate with greater synovial inflammation and joint damage, while sufficient vitamin D levels could exert protective effects [12,31].

## 5. The Epigenetic Role of Vitamin D in RA

The VDR is a ligand-activated transcription factor belonging to the nuclear receptor superfamily, expressed ubiquitously across tissues and cells, with particularly high expression in immune cells. Upon binding to calcitriol in the cytoplasm, the VDR heterodimerizes with the retinoid X receptor (RXR) and translocates to the nucleus, where it binds to vitamin D response elements (VDREs) in promoter and enhancer regions of target genes.

Thus, vitamin D is linked to the regulation of the transcription of over 200 genes involved in immune cell differentiation, cytokine production, antimicrobial peptide synthesis, and immune tolerance [32]. The VDR/RXR dimer interacts with histone acetyltransferases (HATs) to activate gene transcription. It also regulates chromatin accessibility by influencing histone demethylases. Vitamin D signaling is correlated with the methylation of CpG islands in the promoters of genes, including those that regulate their own metabolism (e.g., CYP2R1, CYP27B1, CYP24A1), which can directly impact the body’s vitamin D levels [32,33] (Table 1).

### 5.1. DNA Methylation

A landmark human observational study examining the DNA methylation status of vitamin D pathway genes revealed that CpG islands in the promoter regions of VDR, CYP24A1, and CYP2R1 genes exhibit differential methylation patterns in RA patients compared to healthy controls [6]. The study reported similar overall methylation patterns between RA patients and controls in the promoter regions of these genes (VDR gene 2.39% vs. 2.48%, CYP24A1 gene 16.02% vs. 15.17%, and CYP2R1 2.53% vs. 2.41%) yet revealed a positive correlation between VDR and CYP2R1 methylation intensity and vitamin D levels, specifically in RA-affected participants. Notably, CYP24A1 methylation intensity was significantly higher compared to VDR and CYP2R1 methylation in both groups (*p* < 0.0001), suggesting differential epigenetic regulation of these vitamin D-metabolizing enzymes [6].

Critically, vitamin D-deficient RA patients showed significantly higher CYP24A1 methylation intensity compared to vitamin D-deficient controls (*p* = 0.0104); aberrant epigenetic regulation of CYP24A1 could be hypothesized as a mechanism potentially contributing to impaired vitamin D metabolism in RA [6].

Since CYP24A1 encodes the 24-hydroxylase responsible for inactivating vitamin D metabolites, abnormal methylation-mediated suppression of its expression could be related to reduced catabolism and altered systemic vitamin D levels. These observed findings suggest a possible relationship wherein vitamin D deficiency may be associated with epigenetic alterations in vitamin D-metabolizing genes, potentially involved in a self-perpetuating cycle of dysregulated vitamin D metabolism in RA patients. Additionally, epigenetic alterations affecting VDR expression or function could be linked to reduced capacity of immune cells to respond appropriately to vitamin D signaling, even when circulating levels are adequate [9].

### 5.2. Histone Modifications and Chromatin Remodeling

Histone modifications represent another critical epigenetic mechanism regulating gene expression in response to vitamin D signaling. Recent research has explored the role of histone deacetylase 3 (HDAC3) in regulating VDR promoter activity and vitamin D-responsive gene transcription. A groundbreaking animal study has reported that intestinal butyrate may contribute to ameliorating experimental RA in mice by promoting cortistatin expression via the HDAC3-VDR pathway [34]. The mechanism is associated with a butyrate-induced reduction in HDAC activity in murine intestinal epithelial cells, which has been shown to increase histone acetylation at specific regions (P3 and P4) of the VDR promoter, thereby potentially enhancing VDR transcription and activity. This selective enhancement of VDR expression in intestinal epithelium correlates with cortistatin expression, an anti-inflammatory neuropeptide with potential RA-suppressive properties [34].

The broader implications of histone modification-mediated regulation of vitamin D signaling extend beyond a single pathway to involve orchestrated remodeling of chromatin architecture at multiple vitamin D-responsive genomic loci. Vitamin D-mediated epigenetic changes show an association with histone methylation and acetylation status at promoters and enhancers of genes encoding anti-inflammatory mediators, regulatory T cell differentiation factors, and immune tolerance-promoting molecules [34]. The dynamic interplay between chromatin remodeling complexes, histone-modifying enzymes, and VDR-mediated transcription may contribute to the responsive modulation of immune gene expression in the context of varying vitamin D signaling intensity and immune stimulation status.

### 5.3. MicroRNA Regulation and Vitamin D Signaling

MicroRNAs (miRNAs) represent a distinct layer of epigenetic regulation whereby small non-coding RNAs (approximately 22 nucleotides in length) suppress gene expression through mRNA degradation or translational repression following binding to complementary sequences in target mRNA 3′ untranslated regions. Vitamin D signaling has been shown to regulate the expression of multiple miRNAs that collectively modulate immune response genes, inflammatory pathways, and autoimmunity-related targets. Specifically, vitamin D has been reported to suppress the expression of several pro-inflammatory miRNAs, as shown in vitro on human cells and in mouse and rat model experiments (i.e., miR-155, miR-98-5p, let-7a, miR-149-5p), potentially contributing to a coordinated epigenetic shift toward immune tolerance [35].

### 5.4. VDR Genetic Polymorphisms and Gene–Gene Interactions

While epigenetics encompasses reversible modifications, genetic polymorphisms represent permanent sequence variations that may influence vitamin D signaling capacity and disease susceptibility. Several genetic polymorphisms in the human VDR gene have been identified as potentially modifying vitamin D responsiveness and RA susceptibility. The most studied polymorphisms include the FokI (rs2228570), BsmI (rs1544410), ApaI (rs7975232), and TaqI (rs731236) variants [40]. Research has reported that FokI genotype variations are associated with differences in VDR protein length and transactivation efficiency, with the shorter “f” allele producing a more transcriptionally active receptor [36]. Specific studies investigating VDR polymorphisms in cells from RA populations have observed that carriers of the CT and CC genotypes of rs731236 TaqI are associated with increased RA susceptibility and higher disease activity, while the rs11568820 variant in the VDR promoter region was associated with reduced remission rates in RA patients receiving anti-TNF therapy [37].

A comprehensive investigation of human gene–gene interactions among vitamin D metabolism genes revealed that specific combinations of polymorphisms in CYP2R1, CYP27B1, CYP24A1, and VDR have significant association with both vitamin D levels and RA disease activity [38]. The study found that the GG genotype of rs10877012 (CYP27B1) was associated with hypovitaminosis D (OR = 1.8; *p* = 0.01), while the CT genotype of rs731236 TaqI (VDR) was linked to susceptibility to both RA (OR = 1.9; *p* < 0.01) and high Disease Activity Score 28—Erythrocyte Sedimentation Rate (DAS28-ESR) scores (OR = 3.6; *p* < 0.01). Additionally, FokI and TaqI polymorphisms of the VDR gene in an observational study showed associations with parathyroid hormone (PTH) levels in RA patients, with significant differences among FokI genotypes regarding PTH levels (*p* = 0.009) [39].

Multifactor dimensionality reduction (MDR) analysis examining human gene–gene interactions identified complex epistatic relationships among vitamin D metabolism genes, whereby specific combinations of polymorphisms correlate with more pronounced effects on vitamin D status and RA risk than individual polymorphisms alone. Furthermore, genetic studies of VDR polymorphisms in RA patients undergoing anti-TNF therapy revealed that the rs7975232 A allele was associated with lower vitamin D levels and age at diagnosis (*p* = 0.029 and *p* = 0.017, respectively), yet paradoxically, this same allele was associated with lower DAS28 values after 6 months of therapy and was more common in patients achieving remission (*p* = 0.030 and *p* = 0.004, respectively) [36].

These findings underscore the complexity of genetic contributions of vitamin D in RA pathophysiology, reporting that VDR and vitamin D metabolism gene polymorphisms show an association not only with disease susceptibility and activity, but also with the response to both vitamin D supplementation and biologic therapies such as anti-TNF agents.

## 6. Evidence for the Effect of Vitamin D on Seasonal Changes in RA Clinical Activity

### 6.1. Circannual Rhythm of Vitamin D Synthesis and Bioavailability

The production of vitamin D from cutaneous 7-dehydrocholesterol is fundamentally dependent upon exposure to UVB radiation, which exhibits marked seasonal variation related to solar zenith angle that in turn depends on latitude, season, time of day, and atmospheric conditions [13,17].

Furthermore, the season of RA onset can influence disease severity; winter or spring onset has been linked to fatigue, faster radiographic progression, and lower remission rates within a year compared to summer or autumn onset [41] (Table 2).

A detailed retrospective analysis of 101 RA patients receiving methotrexate or leflunomide therapy and vitamin D supplementation (2000 IU daily) revealed significant seasonal variation in 25(OH)D concentration (*p* = 0.004), with mean summer levels of 50.63 ± 17.73 ng/mL in leflunomide-treated patients and 34.73 ± 14.04 ng/mL in methotrexate-treated patients [42]. The study further demonstrated a correlation between 25(OH)D levels and RA duration specifically in the summer population (r= −0.64; *p* < 0.05), while in other seasons (r= 0.14; *p* > 0.05), suggesting that seasonality can be linked to vitamin D status differently depending on disease duration and pharmacological treatment.

### 6.2. Seasonal Patterns in RA Disease Activity and Inflammatory Markers

**Figure 2:**
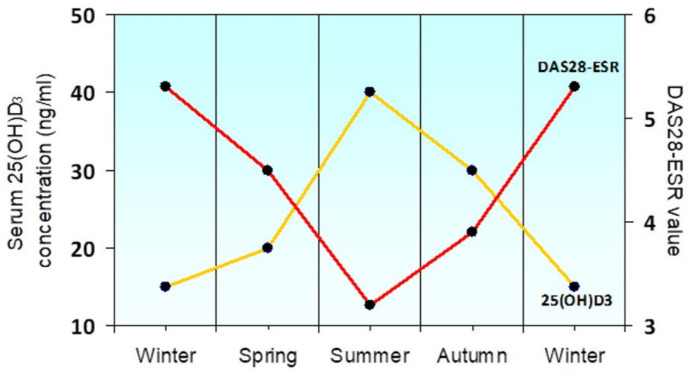
Seasonal variations in serum concentration of 25(OH)D_3_ and correlation with RA disease activity measured by DAS28-ESR score. Legend: 25(OH)D_3_ levels and DAS28-ESR values reflect actual measurements found on average in the studies analyzed and were adapted for fitting in the figure. The graph shows seasonal correlation across a full year of observation. Abbreviations: 25(OH)D_3_: 25-hydroxyvitamin D_3_; DAS28-ESR: Disease Activity Score-28—Erythrocyte Sedimentation Rate.

Several epidemiological studies have documented seasonal variation in RA disease activity, with generally higher disease activity during winter months and lower activity during summer months, though findings have shown some inconsistency regarding specific patterns [12,45,46,47,48] (Figure 2).

A comprehensive analysis of 12,839 Japanese RA patients examining seasonal patterns of disease activity found that mean DAS28-C Reactive Protein (DAS28-CRP) scores were highest in spring and lowest in autumn (*p* < 0.05), with fall associated with significantly higher remission rates compared to other seasons [14]. The distribution of affected joints also showed seasonal variation, with upper extremity involvement showing the most pronounced seasonal pattern. Furthermore, the study demonstrated seasonal variation in functional disability and systemic inflammation markers, with spring and winter associated with higher disease activity scores compared to autumn. Therefore, seasonality of vitamin D synthesis in the skin might be a contributing factor for the seasonal variation in RA activity, since vitamin D can modulate both innate and adaptive immunity.

Somewhat contradictory findings were reported in a study of 71 RA patients in which bi-seasonal measurements of serum 25(OH)D vitamin and disease activity assessments using DAS28 revealed no significant correlation between serum vitamin D levels and disease activity scores during either winter or summer (*p* > 0.05) and no significant difference in DAS28 scores between winter and summer seasons [43]. However, the study is limited by a low number of patients and consistent vitamin D supplementation throughout the year. These conflicting results highlight the heterogeneity in published studies and the need for larger, more rigorously controlled investigations with standardized methodology to clarify the relationship between seasonal vitamin D fluctuations and RA disease activity variation.

The *Étude et Suivi des POlyarthrites Indifférenciées Récentes* (ESPOIR) cohort study, which enrolled a total of 813 patients with early arthritis, of which 645 patients with RA were considered for the analysis, has highlighted that vitamin D deficiency was associated with more active and severe disease at baseline and may predict disability and radiographic progression over 1 year in early RA patients [41]. In particular, the vitamin D level was <10 ng/mL (deficiency, group 1), 10–29.9 ng/mL (low level, group 2), and ≥30 ng/mL (normal, group 3) for 114 (17.7%), 415 (64.54%), and 114 (17.7%) patients, respectively. At baseline, DAS28-ESR and the Health Assessment Questionnaire-Disability Index (HAQ-DI) were higher with vitamin D deficiency compared with groups 2 and 3 combined (*p* = 0.007 and *p* = 0.001, respectively), as was the mean van der Heijde modified total Sharp score (mTSS), but not significantly (*p* = 0.076) [41].

### 6.3. Geographic Latitude and Seasonal Fluctuations

Geographic latitude significantly influences the amplitude and timing of seasonal vitamin D fluctuations, as well as the overall prevalence of vitamin D deficiency within populations.

A meta-analysis of 24 studies involving 3489 RA patients revealed a negative relationship between serum 25(OH)D levels and disease activity (DAS28: r = −0.13, 95% CI: −0.16 to −0.09), and notably, latitude-stratified subgroup analysis yielded a relatively stronger negative correlation between 25(OH)D and DAS28 in low-latitude areas (r = −0.26, 95% CI: −0.35 to −0.16) compared to high-latitude regions [44].

This finding suggests that the association between vitamin D status and RA disease activity appears more pronounced in regions where vitamin D bioavailability is less constrained by seasonal and geographic factors. In regions at high latitudes, alternative mechanisms may partially compensate for low serum vitamin D levels, or populations may have been selected for genetic factors conferring relative vitamin D sufficiency.

A multicenter study (COMOrbidities in Rheumatoid Arthritis [COMORA] study) evaluating vitamin D status in 1413 RA patients across 15 countries found that vitamin D deficiency (25(OH)D < 20 ng/mL) occurred in 8.5% of patients and insufficiency (20–30 ng/mL) in 54.6%, with marked geographic variation [49]. The study demonstrated that vitamin D status was associated with various patient characteristics including age, BMI, educational level, disease activity, glucocorticoid dosage, and comorbidities, for instance osteoporosis.

Furthermore, the absence of vitamin D supplementation was strongly related to a higher prevalence of deficiency (*p* < 0.001), suggesting that geographic regions with lower natural sun exposure and vitamin D bioavailability would particularly benefit from population-level vitamin D supplementation strategies.

## 7. Vitamin D Administration in RA

### 7.1. Correction of Vitamin D Deficiency and Skeletal Indications

The optimal vitamin D supplementation regimen for RA patients remains incompletely defined, with considerable heterogeneity in published trials regarding dose magnitude, treatment duration, and baseline patient selection (Table 3).

Contemporary supplementation regimes studied in RA populations have ranged from 2000 IU daily (vitamin D_3_) to 50,000 IU weekly (the latter is not fully suggested), with treatment durations ranging from 12 weeks to 12 months [54,55]. The dose–response relationships identified in systematic reviews seem to suggest that higher doses (>50,000 IU weekly) might be correlated with more pronounced disease activity improvements compared to lower doses, though optimal dosing strategies considering efficacy, safety, individual patient factors, and baseline vitamin D status remain incompletely characterized.

However, it should be noted that excessively high single doses of vitamin D supplements induce CYP24A1, which promotes the catabolism and elimination of vitamin D metabolites [56]; therefore, high single dosages during a month appear to be less efficient.

A vitamin D supplementation guideline recommending personalized approaches suggested that optimal 25(OH)D concentrations range from 50 to 70 ng/mL (125–175 nmol/L) for general health maintenance, with potentially higher targets (60–90 ng/mL) for patients with autoimmune diseases including RA [20,54]. For RA patients identified with vitamin D deficiency or insufficiency, supplementation dosages of 1000–2000 IU daily or 4000–7000 IU daily, with periodic reassessment of serum 25(OH)D levels to verify adequate repletion and maintenance, represent reasonable current approaches. However, the recognition that responses to supplementation are heterogeneous and can be potentially modulated by VDR genetic polymorphisms, baseline vitamin D status, disease activity severity, and concurrent medications suggests that personalized or stratified supplementation approaches might be more effective than one-size-fits-all strategies [20,54]. However, this hypothesis requires prospective clinical validation.

Of note, with specific regard to osteoporosis, a common multifactorial comorbidity associated with RA, adequate serum vitamin D levels enhance intestinal calcium absorption by upregulating the expression of the vitamin D-dependent calcium-binding protein (calbindin-D9k) in the intestinal epithelium. At the systemic level, sufficient vitamin D status also promotes osteoblast activity, reduces osteoclast-mediated bone resorption, and prevents secondary hyperparathyroidism, thereby limiting excessive bone turnover [57]. A large meta-analysis including 57 studies further confirmed that adequate serum vitamin D concentrations are associated with a lower risk of osteoporosis in patients with RA (OR = 0.88), while vitamin D and/or calcium supplementation were associated with an even greater protective effect (OR = 0.49) [58].

### 7.2. Potential Immunomodulatory Effects in RA Treatment and in Prevention of the Disease

Systematic reviews and meta-analyses have synthesized evidence from randomized controlled trials, examining the effects of vitamin D supplementation on RA disease activity and clinical outcomes. A comprehensive meta-analysis of 11 studies involving patients with RA demonstrated that vitamin D supplementation was related to significant improvements in multiple disease activity markers compared to placebo or standard care [16]. The results showed major influence of vitamin D supplementation on DAS28 (Weighted Mean Difference [WMD]: −0.83, 95% CI: −1.38 to −0.28, *p* < 0.001), CRP levels (WMD: −0.24, 95% CI: −0.45 to −0.03, *p* = 0.03), and ESR levels (WMD: −4.08, 95% CI: −4.67 to −3.50, *p* < 0.001). The GRADE assessment of evidence quality indicated moderate certainty for all outcomes except serum vitamin D levels, which demonstrated high certainty of evidence. Notably, the analysis found non-significant effects of supplementation on health assessment questionnaire (HAQ) scores and visual analog scale (VAS) pain scores in the pooled analysis, though subgroup analyses revealed differential effects depending on baseline vitamin D status and supplementation dosage [16].

A dose–response meta-analysis of vitamin D supplementation effects revealed heterogeneous responses based on vitamin D dose magnitude and treatment duration [15]. The study found that vitamin D supplementation significantly reduced pain-VAS scores (WMD = −1.30, 95% CI: −2.34 to −0.27, *p* = 0.01), DAS28–CRP (WMD = −0.58, 95% CI: −0.86 to −0.31, *p* < 0.0001), and DAS28–ESR (WMD = −0.58, 95% CI: −0.86 to −0.31, *p* = 0.0001). A subgroup analysis stratified by vitamin D dose showed that doses exceeding 50,000 IU may be associated with more significant improvements in VAS and DAS28 compared to lower doses, though substantial heterogeneity persisted among studies [15]. Confounding factors potentially explaining heterogeneity included baseline vitamin D levels, patient age, dietary vitamin D intake, time of year when measurements were obtained, sun exposure, drug interactions, effect dosage, and statistical power of individual studies.

Another systematic review and meta-analysis specifically focusing on clinical and laboratory biomarkers found that vitamin D supplementation significantly improved VAS scores (*p* < 0.001), serum vitamin D levels (*p* < 0.001), and CRP levels (*p* < 0.001), but did not significantly improve other outcomes like DAS28 or ESR [50].

The substantial heterogeneity across these outcomes (I^2^ values ranging from 84.1% to 97.4%) suggests that study-level characteristics including patient selection criteria, baseline disease activity, concurrent medications, vitamin D dose and duration, and measurement techniques significantly influence reported outcomes.

Recently, the VITamin D and OmegA-3 TriaL (VITAL) study assessed whether vitamin D or omega-3 supplementation prevented incident autoimmune diseases, such as RA, polymyalgia rheumatica, autoimmune thyroid disease, and psoriasis, in 25,871 participants (median age 67 years) followed for a median of 5.3 years [50]. Vitamin D3 supplementation reduced confirmed autoimmune disease by 22% (Hazard Ratio [HR] 0.78, 95% CI: 0.61 to 0.99, *p* = 0.05). The effect appeared stronger when including probable cases (HR 0.85, 95% CI: 0.74 to 0.99). Omega-3 fatty acids showed a non-significant 15% reduction in confirmed autoimmune disease (HR 0.85, 95% CI: 0.67 to 1.08, *p* = 0.19), though the effect became significant when including probable cases (HR 0.87, 95% CI: 0.77 to 0.99) [51].

A prospective follow-up study examined whether benefits persisted two years after stopping supplementation in participants from the VITAL study who remained autoimmune disease-free during the active trial period [52]. During the 2-year post-intervention period, vitamin D continued to show protective effects with a 22% reduction in incidence of autoimmune disease (HR 0.78, 95% CI: 0.55 to 1.10), though this did not reach statistical significance. Omega-3 fatty acids showed sustained benefit with a 28% reduction (HR 0.72, 95% CI: 0.51 to 1.01). When combining the active treatment period with the 2-year follow-up (total ~7 years), vitamin D reduced autoimmune disease incidence by 15% (HR 0.85, 95% CI: 0.70 to 1.04) and omega-3 levels by 18% (HR 0.82, 95% CI: 0.68 to 0.99). The sustained effects after supplementation cessation raise the possibility of disease-modifying effects rather than merely suppressing symptoms during active treatment [52].

At last, a larger epidemiological study analyzing data from the National Health and Nutrition Examination Survey (NHANES) including 2290 RA patients found a strong negative correlation between 25(OH)D levels and all-cause mortality (HR: 0.91 per 10 nmol/L increase, 95% CI: 0.87 to 0.96) [52]. Cause-specific analysis revealed that higher 25(OH)D levels were associated with reduced mortality from heart disease (HR: 0.88, 95% CI: 0.82 to 0.95) and malignant neoplasms (HR: 0.86, 95% CI: 0.79 to 0.94). An age-stratified analysis suggests that vitamin D sufficiency may be increasingly important for long-term survival, especially for older RA populations [53].

## 8. Discussion and Conclusions

The present narrative review highlights the role of vitamin D, which should be mainly considered a secosteroid hormone with extensive immunomodulatory activity. The evidence confirms that vitamin D deficiency is highly prevalent in RA patients and correlates positively with disease activity, inflammatory markers, and early radiographic damage [11,41,59,60]. Notably, the emerging picture suggests that this deficiency is not simply a consequence of systemic inflammation or determined by a certain kind of lifestyle (i.e., low UVB body exposure or micronutrient intake insufficiency) but may contribute to ongoing immune dysregulation through complex epigenetic and seasonal mechanisms [60,61,62].

At the core of vitamin D’s proposed activity in RA lies its potential capacity to modulate both innate and adaptive immune responses. Vitamin D, by promoting macrophage polarization toward the anti-inflammatory M2 phenotype and suppressing Th1/Th17 responses in favor of regulatory B and T cells, can be associated with the control of autoimmune responses [20,24].

A novel aspect highlighted in this review is its epigenetic role; in fact, by interacting with its receptor (VDR), vitamin D modulates chromatin accessibility and DNA methylation. For instance, the aberrant hypermethylation of the *CYP24A1* gene observed in deficient patients suggests a possible vicious cycle where deficiency may be linked to epigenetic alterations in vitamin D metabolism, making serum repletion more challenging [6].

Furthermore, the recognition of seasonal variation in RA disease activity has significant implications for clinical management, suggesting that seasonal adjustment of vitamin D supplementation correlated with disease activity remains a highly considerable hypothesis that warrants further prospective validation before routine clinical implementation [62].

However, an important aspect that should be considered when interpreting the available evidence is the inherent complexity of serum 25(OH)D as a biomarker of vitamin D status. Measured concentration may be influenced by assay variability, seasonal intra-individual variability, and physiological factors such as obesity, which alters vitamin D distribution and bioavailability [63,64]. Moreover, total circulating 25(OH)D may not fully reflect the biologically active fraction, as growing evidence suggests that free or bioavailable vitamin D may better represent vitamin D status in specific clinical settings [65]. The interpretation of serum 25(OH)D levels may also be complicated by concomitant treatments frequently used in patients with autoimmune diseases, including glucocorticoids and biologic therapies, which can affect vitamin D metabolism and immune function [20]. These factors should be considered when comparing studies and interpreting associations between vitamin D status and disease activity.

Similarly, an important limitation in interpreting the association between vitamin D deficiency and RA disease activity is the potential for reverse causation. Patients with more severe disease often experience reduced mobility, lower levels of physical activity, increased disability, and less sunlight exposure, all of which may contribute to lower serum 25(OH)D concentrations independently of any causal role of vitamin D in disease pathogenesis. Consistent with this interpretation, prospective studies evaluating pre-diagnostic vitamin D status have not demonstrated a clear association with subsequent RA risk, highlighting the need for Mendelian randomization studies and adequately powered randomized trials to clarify causality [66].

Of note, the most recent recommendations for the management of RA do not recognize vitamin D as DMARD. This is mainly because randomized clinical trials remain heterogeneous, as do their reported effect sizes. The evidence accumulated to date in humans is still inconclusive, and further studies are needed to clarify its therapeutic role [67].

Nevertheless, the relative contribution of seasonal vitamin D fluctuations versus other seasonal factors (UV exposure, infectious agents, environmental allergens, temperature changes affecting physical activity, lifestyle, diet, air pollution) remains incompletely investigated. Available evidence does not allow these effects to be clearly disentangled. A recent narrative review exploring environmental influences on RA status emphasized that while seasonality influences disease activity, the mechanisms underlying this relationship are multifactorial and likely involve vitamin D in seasonal variation and dietary management based on seasonal and latitudinal food availability [68,69,70,71].

Despite the documented seasonal variation in RA disease activity, implementing season-based treatment modifications requires careful consideration of specific diets and lifestyle, and in particular, further prospective validation before integration into routine clinical practice. Although observational data consistently support an inverse association between vitamin D status and RA disease activity, the heterogeneity in published studies regarding the strength and consistency of seasonal effects suggests that the relationship between vitamin D and seasonal RA clinical activity is modulated by multiple variables.

Ultimately, further studies with standardized methodologies and larger patient cohorts across diverse geographic regions are ongoing to clarify optimal strategies for leveraging seasonal vitamin D fluctuations in RA management and the roles in other autoimmune rheumatic diseases [11].

## Figures and Tables

**Table 1:** Selected recent original studies and reviews on epigenetic and genetic modifications upon vitamin D in RA.

Study Authors (Year) [Ref.]	Study Design	Molecular Mechanism	Key Genetic Target	Primary Associations
Puncevičienė et al. (2022) [6]	Observational study Human cohort (RA patients and healthy controls)	DNA methylation	VDR, CYP24A1, CYP2R1	Promoter CpG methylation of vitamin D pathway genes is related to RA, with particularly high CYP24A1 methylation in vitamin D-deficient RA patients, hypothesizing aberrant epigenetic control of vitamin D metabolism.
Liao et al. (2025) [34]	Experimental study Animal model (mouse)	Histone modification/HDAC inhibition	HDAC3, VDR promoter, cortistatin	Intestinal butyrate associates with inhibiting HDAC3, increasing histone acetylation at the VDR promoter, upregulating VDR and cortistatin, and correlating with improvement in experimental RA through an HDAC3–VDR–cortistatin pathway.
Gotelli et al. (2024) [35]	Review of Experimental studies In vitro (human) and animal models (mouse and rat)	microRNA regulation	miR-155, miR-98-5p, let-7a, miR-149-5p	Vitamin D signaling has been shown to downregulate several pro-inflammatory miRNAs, potentially contributing to shifting the miRNA profile toward immune tolerance and dampening inflammatory pathways relevant to RA.
Wielińska et al. (2024) [36]	Observational study Human cohort (patients)	VDR genetic polymorphisms	VDR genetic variants (FokI, BsmI, ApaI, TaqI)	Functional VDR variants, especially FokI and ApaI/TaqI, correlate with alteration of receptor activity, vitamin D levels, and RA disease activity and remission rates during anti-TNF therapy, hypothesizing that VDR genotype can modulate clinical response.
Latini et al. (2024) [37]	Observational study Human (RA patients)	VDR promoter polymorphism	VDR rs11568820 (promoter variant)	The rs11568820 promoter variant is associated with a lower probability of remission in RA patients on anti-TNF therapy; VDR transcriptional regulation can be linked to treatment response.
Campos-López et al. (2025) [38]	Observational study Human (RA patients)	Gene–gene interactions in vitamin D metabolism	CYP2R1, CYP27B1, CYP24A1, VDR	Combinations of polymorphisms across vitamin D metabolism genes (e.g., CYP27B1 rs10877012, VDR rs731236) are more strongly associated with vitamin D status and RA risk/activity than single variants alone, highlighting epistatic effects.
Ahmad et al. (2023) [39]	Observational study Human (RA patients)	VDR polymorphisms and endocrine axis	VDR genetic variants (FokI, TaqI), PTH	VDR FokI and TaqI polymorphisms are associated with differences in parathyroid hormone levels in RA, suggesting VDR genetic variation could influence the broader vitamin D–PTH–bone metabolism axis.

Abbreviations: Ref: reference; VDR: vitamin D receptor; CYP24A1: cytochrome P450 family 24 subfamily A member 1; CYP2R1: cytochrome P450 family 2 subfamily R member 1; RA: rheumatoid arthritis; HDAC: histone deacetylase; miRNA: microRNA; TNF: tumor necrosis factor; CYP27B1: cytochrome P450 family 27 subfamily B member 1; PTH: parathyroid hormone.

**Table 2:** Evidence on seasonal vitamin D levels and RA activity patterns in humans.

Study Authors (Year) [Ref.]	Study Design	Sample Size (Patients)	Geographic Location	Vitamin D Peak	RA Activity Pattern	Vitamin D Supplementation Status	Correlation with Vitamin D
Cieślewicz et al. (2024) [42]	Observational Retrospective	101	Poland	Summer	Seasonal variation observed (*p* = 0.004)	2000 IU daily	No significant correlation
Mori et al. (2019) [14]	Observational Retrospective	12,839	Japan	Not specified	Highest in spring; lowest in autumn	n/a	Seasonal pattern noted
Yazmalar et al. (2013) [43]	Observational Prospective	71	Turkey	Summer/Fall	No significant winter–summer difference	n/a	No correlation (*p* > 0.05)
Cutolo et al. (2006) [12]	Observational Prospective (PIVOTAL study on this topic)	54 Italian 64 Estonian	Italy and Estonia	Summer	Negative correlation with disease activity (DAS28) found in summer only in IP (r = −0.57, *p* < 0.0001) and in winter in EP (r = −0.40, *p* < 0.05)	n/a	Seasonal pattern noted Stronger in Southern Europe
Lin et al. (2016) [44]	Meta-Analysis	3489	Multiple regions	Varies by latitude	Negative correlation with disease activity (DAS28) (r = −0.13, 95% CI −0.16 to −0.09)	n/a	Stronger in low latitudes

Abbreviations: IU: international unit; n/a: not applicable-not available; DAS28: disease activity score-28; IP: Italian patients; EP: Estonian patients.

**Table 3:** Evidence on vitamin D deficiency and supplementation studies in RA.

Study Authors (Year) [Ref.]	Population	Geographic Location	Vitamin D Deficiency Rate	Primary Findings
Ranjbar et al. (2025) [16]	1129 participants (12 RCTs)	Multiple countries	40–60%	Vitamin D supplementation improved DAS28, CRP, and ESR
Al-Saoodi et al. (2024) [15]	3049 RA patients (11 RCTs)	Multiple countries	50–70%	Significant reduction in VAS and DAS28-CRP with supplementation
Cieślewicz et al. (2024) [42]	101 RA patients on MTX/LEF	Poland	28.7%	Significant seasonal variation (*p* = 0.004); no correlation with activity
Puncevičienė et al. (2022) [6]	76 participants (35 RA, 41 controls)	Lithuania	Similar patterns	CYP24A1 methylation higher in vitamin D-deficient RA patients
Mouterde et al. (2020) [41]	645 RA patients	France	17.7%	Vitamin D deficiency was associated with more active and severe disease at baseline and may predict disability and radiographic progression over 1 year in early RA patients
Khatirnamani et al. (2025) [50]	1390 participants (12 RCTs)	Multiple countries	12.5%	Vitamin D supplementation significantly improved VAS pain scores, serum 25(OH)D levels, and CRP (all *p* < 0.001)
Hajjaj-Hassouni et al. (2017) [49]	1413 RA patients	Multiple countries	8.5%	Absence of vitamin D supplementation was strongly related to higher prevalence of deficiency (*p* < 0.001)
Hahn et al. (2022) [51]	25,871 participants	USA	12.9%	Vitamin D supplementation for 5 years, with or without omega-3 fatty acids, reduced autoimmune disease by 22%
Costenbader et al. (2024) [52]	21,592 participants	USA	n/a	Over the 2-year post-trial period (VITAL study), protective effects of vitamin D on incident autoimmune disease diminished and were no longer statistically significant
Feng et al. (2024) [53]	2290 RA patients (NHANES)	USA	30–40%	Lower 25(OH)D inversely associated with all-cause mortality

Abbreviations: RCTs: randomized controlled trials; CRP: C-reactive protein; ESR: erythrocyte sedimentation rate; VAS: visual analog scale; MTX: methotrexate; LEF: leflunomide; 25(OH)D: 25-hydroxyvitamin D; USA: United States of America; NHANES: national health and nutrition examination survey.

## Data Availability

No new data was generated in this review.
